# Examining the mental health trajectories of children and adolescents: a cross-cohort analysis

**DOI:** 10.1017/S0033291724001624

**Published:** 2024-11

**Authors:** Fiona McNicholas, Blanaid Gavin, Ruth Sellers, Iris Ji, Xiaoning Zhang, Wendy V Browne, Gordon Harold

**Affiliations:** 1 School of Medicine, University College Dublin, Ireland; 2 CHI Crumlin Dublin 12, Ireland; 3 CAMHS Lucena Clinic Rathgar Dublin 6, Ireland; 4 University of Cambridge, UK; 5 Andrew and Virginia Rudd Research and Professional Practice Centre, Cambridge, UK

**Keywords:** child and adolescent mental health trajectories, cohort longitudinal studies, international cohort comparisons, mental health service and policy planning

## Abstract

**Background:**

Epidemiological samples provide opportunity to understand the development of mental health trajectories to better understand whether such epidemiological data can help to plan and modify service delivery for youth mental health. Variation between countries is not well understood and thus applying evidence from other countries to national strategies limits support service policy and planning. We therefore examine developmental patterns of youth mental health across different countries using the Growing Up in Ireland (GUI) Cohorts, with comparison to existing UK longitudinal cohort data (Millennium Cohort Study, MCS; Growing up in Scotland, GUS).

**Methods:**

Youth mental health problems within each cohort across development (5–17/18 years) were assessed using parent reported Strengths and Difficulties Questionnaire (SDQ) scores. Using latent growth curve analyses, we examined trajectories of emotional, conduct, and hyperactivity problems for boys and girls, separately for each cohort.

**Results:**

Across cohorts, we observed similar developmental patterns for emotional, conduct, and hyperactivity problems. However, the GUI emotional problems in Ireland emerged earlier than in the UK. By adolescence, GUI emotional scores were similar to the UK, suggesting that the differences in emotional problems between the ROI and UK had narrowed by adolescence. Covariates also had different associations with youth mental health trajectories across cohorts.

**Conclusions:**

Utilizing multiple nationally representative cohort longitudinal datasets can help inform clinically meaningful conclusions and potential recommendations on population level multi-tiered service needs and development in the area of child and adolescent mental health support and future provision.

Despite the growing financial constraints in most countries and the rising costs of health care, strong economic arguments for investment in mental health services remain. It is anticipated that the global cost of mental illness in terms of poor health and lost productivity will cost $6 trillion by 2030 (Health, 2020). A recent UK report (McDaid et al., 2022) estimates current and conservative costs of 5% of the UK's GDP or £117.9 billion per year. The argument for investment is easily justified by evidence of significant returns, $4 for every $1 invested in the case of adults with depression and anxiety (Health, 2020). Given the childhood origins of most of adult mental illness (Jones, 2013; Kessler et al., 2005), potentially greater returns may accrue when such investments are made earlier in childhood. Despite this evidence, service development and investment lag behind, leading in 2020, to an urgent WHO global call to ‘Move for mental health: Let's invest’ (Health, 2020).

Focusing on child and adolescent mental health service supports and development is also pertinent as there is indication that prevalence rates of adolescent mental health problems are increasing internationally. UK surveys show an increase in mental health difficulties (Collishaw, 2015; NHS Digital, 2017, 2020; O'Neill & Rooney, 2018; Patalay & Gage, 2019; Pitchforth et al., 2019; Sadler et al., 2018) particularly for emotional difficulties (e.g. anxiety and depression) amongst females (Collishaw, 2015; Patalay & Gage, 2019). Similar increased rates of mental health difficulties have been reported internationally (Collishaw, 2015; Lu, 2019). Whether such increases in prevalence are observed in the Republic of Ireland (ROI) is more difficult to establish, but evidence from a sample of Irish adolescents suggests increases in internalizing problems, with higher rates for females (Cannon, Coughlan, Clarke, Harley, & Kelleher, 2013; Dooley, Fitzgerald, & Giollabhui, 2015).

There is well-established evidence that many mental health challenges first manifest in childhood, and then increase across adolescence (Merikangas, Nakamura, & Kessler, 2022). However, there is much heterogeneity in the development of mental health difficulties, including age at onset and clinical course. With regards to externalizing problems, some studies suggest that hyperactivity and conduct problems both decrease across childhood and adolescence, with boys generally reporting higher symptoms than girls (Nagin & Tremblay, 2001). However, a cross-national study suggested that whilst boys reported higher levels of disruptive behaviors, patterns of behavior differed between countries, with disruptive behaviors and aggression in boys showing patterns of growth in US samples, stability in New Zealand samples, and declines in Canadian samples (Broidy et al., 2003). Findings from a UK study also suggest patterns of growth across early adolescence (Bains & Gutman, 2021). With regards to emotional problems, recent studies from the UK (Kwong et al., 2019) and US (Crosnoe & Thorpe, 2022; Meadows, Brown, & Elder, 2006) suggest that overall, symptoms tend to increase across adolescence but that girls tend to have an earlier onset with higher levels of symptoms across development compared to boys.

Overall, however, less is known about differences in developmental patterns across different countries. For example, in a recent study the extent of gender and social adversity differences were not similar between countries over time, nor were the influences of various risk factors such as parental education and employment (Terhaag, Fitzsimons, Daraganova, & Patalay, 2021). Thus, developmental trajectories may not be the same across different developmental stages when assessed in different countries/contexts. Any differences between countries may reflect real differences in psychopathology, as well as contextual and cultural differences. However, it is important to consider that many studies across different countries use different methodologies, making it difficult to compare prevalence rates and developmental patterns in youth mental health (Lynch, McDonnell, Leahy, Gavin, & McNicholas, 2022). Understanding such differences can be important for planning of services and service development based on population need, but recognized variations between countries in prevalence rates and mental health trajectories over time necessitates caution if extrapolating rates for other studies to advise on service policy and planning.

Alongside increases in mental health, there has also been an upward trend of referrals to, and attendances at, Child and Adolescent Mental Health Services (CAMHS) reported in high-income countries (Collishaw, 2015) including the US (Olfson, Druss, & Marcus, 2015), England (Crenna-Jennings & Hutchinson, 2018; Hickling, Dabrowski, & Williams, 2023), Sweden (Kosidou et al., 2010), and Ireland (McNicholas et al., 2021). However, findings are not consistent, with other studies suggesting that, whilst mental health difficulties increased, use of mental health services remained stable (Lu, 2019). Factors contributing to increased (or reduced) demand for specialist mental health services are complex. It may be that increased demand reflects increased prevalence of psychopathology among youth (as noted above). Alternative explanations may include increased referrals due to a lack of alternative lower-level tiered services, such as community counselling, psychological or social support, educational or disability services. Furthermore, the majority of children with mental health disorders and impairment do not receive clinical diagnosis or mental health services, therefore clinical records and referral data alone cannot tell us about the population prevalence of mental health problems (Collishaw & Sellers, 2020; Merikangas et al., 2022).

Whilst evidence suggests that there is growing demand for services, it is essential that efforts are redoubled to explore existing datasets to determine to what extent, if any, data can be effectively utilized to better estimate population prevalence of mental health problems and inform clinically meaningful conclusions on population level multi-tiered service need. Longitudinal epidemiological cohort studies provide opportunity to examine the prevalence and developmental course of mental health difficulties across childhood and adolescence (Costello, Burns, Angold, & Leaf, 1993; Ford, 2008). Understanding differences in development between cohorts is useful to inform country-specific services development organized on the basis of need (Coughlan & Doyle, 2015; Lynch et al., 2022). The aim of this study was therefore to examine the mental health trajectories of children and adolescents in three representative cohort samples, using very similar methodology, spanning the UK (Millennium Cohort Study [MCS]; Growing up in Scotland [GUS]) and ROI (Growing Up in Ireland [GUI]) to understand the development of mental health trajectories across childhood and adolescence. By considering any systematic differences between cohorts, we can better understand whether such epidemiological data can help to plan and modify service delivery for youth mental health.

## Method

The current study used four longitudinal cohorts: GUI Child and Infant cohorts, the MCS, and GUS. In each cohort, child mental health problems were assessed at each wave, spanning childhood and adolescence (see Table 1).

**Table 1.** Year and age of SDQ assessments per cohort study

	SDQ_T1		SDQ_T2		SDQ_T3		SDQ_T4		SDQ_T5	
	Age	Year	Age	Year	Age	Year	Age	Year	Age	Year
GUI-08	5	2012/13	7/8	2015/16	**9**	2016/17				
GUI-98					**9**	2007	13	2011	17/18	2015
MCS	5	2006	**7**	2008	11	2012	14	2015	17	2018
GUS	6	2010	**8**	2012	10	2015/15	12	2017/18	14	2019/20

*Note.* The ages of demographics are bolded in the table.

### Participants and study design

#### Cohorts I and II, growing up in Ireland (GUI) study

GUI is a national longitudinal study of children (McNamara, Murray, & Williams, 2019). The study includes two cohorts of children, one from 9 years (Cohort 98, formerly the ‘Child Cohort’), and the second from 9 months (Cohort 08, formerly ‘Infant Cohort’). Data collection for both samples started in 2008. Cohort 98 consisted of over 8500 children aged 9 years. Children were eligible if they were born between November 1, 1997 and October 31, 1998 and were selected using a two-stage sampling method within the school system. Children were followed up at age 13, and 17/18 years. For Cohort 08, families were selected from the Child Benefit Register and were born between December 1, 2007 and June 30, 2008. Surveys were completed with over 11 000 caregivers aged 9 months, representing almost a quarter of all births in Ireland during that time. Follow-up waves were conducted at ages 3, 5, 7/8 (postal), and 9 years. Analyses included existing sample weights to adjust for attrition and ensure the structure of the complete sample was representative of the sample population (see Murray, Williams, Quail, Neary, & Thornton, 2015; Quail, Williams, McCrory, Murray, & Thornton, 2011). The study received ethical approval from the Research Ethics Committee of the Office for the Minister for Children and Youth Affairs in Ireland. Further information is available on the website: http://www.esri.ie/growing-up-in-ireland/.

#### Cohort III, millennium cohort study (MCS)

MCS is a large-scale longitudinal birth cohort of children (*N* = 19 519) born between September 2000 and January 11, 2002 in England, Wales, Scotland, and Northern Ireland (Dex & Joshi, 2004; Plewis, Calderwood, Hawkes, Hughes, & Joshi, 2007). For the current study, data were employed from age 5 years to age 17/18 years (University of London, 2021, 2022a, 2022b, 2022c, 2023). The MCS design over-sampled areas with higher proportions of ethnic minorities, areas of high child poverty, and households in Scotland and Wales. In addition, there was some selective attrition (Wadsworth et al., 2003). Therefore, analyses included existing sample weights designed to adjust both for measured predictors of non-response and to correct for sample stratification (see Hansen, 2014; Plewis et al., 2007) to ensure results were representative of the UK population. Ethical approval for MCS was obtained by the London Multi-Centre Research Ethics Committee. Further information is available on the website: http://www.cls.ioe.ac.uk/.

#### Cohort IV: Growing up in Scotland (GUS) study

We employed data from Birth Cohort 1 of the GUS (Anderson et al., 2007; ScotCen Social Research, 2022). This cohort includes 5217 children born between June 2004 and May 2005. Families were selected at random from Child Benefit records. Sample weights provided were used for the analyses to adjust for non-random non-response bias, and for unequal probability of selection for some children (Bradshaw, Corbett, & Tipping, 2011; Bradshaw, Tipping, Marryat, & Corbett, 2007). For the current study, data were employed from age 6 years. Data were collected biennially until the children were in their first year of secondary school (age 12 years). Ethical approval for GUS was obtained from Scotland Research Ethics Committee. Further information is available on the website*:* https://growingupinscotland.org.uk/

### Measures

#### Child mental health

Child mental health difficulties were assessed in each cohort using the Strengths and Difficulties Questionnaire (SDQ; Goodman, 2001), a 25-item screening questionnaire, validated for both general population and clinical samples (Stone, Otten, Engels, Vermulst, & Janssens, 2010). The SDQ contains five subscales. We focused on the three clinical subscales (emotional problems, conduct problems, and hyperactivity problems). Each subscale was calculated by adding responses to five items for that subscale. Each item is scored 0 ‘not true’, 1 ‘somewhat true’ or 2 ‘certainly true’. SDQ scores were assessed using main parent report (usually the mother).

#### Covariates

We considered a number of covariates that have been associated with youth mental health and/or access to mental health services and supports, including household composition (single parent with one or two children; single parent with three or more children; two parents with one or two children; two parents with three or more children; Vella, Gardner, Swann, and Allen, 2019), region type (rural/urban; Fontanella et al., 2015), income (population quintiles within each cohort; Deighton et al., 2019; Hodgkinson, Godoy, Beers, and Lewin, 2017; Vella et al., 2019), and whether the child had special education needs or learning difficulties (yes/no; Deighton et al., 2019). The years when the covariates were measured are noted under Table 2.

**Table 2.** Demographics across cohorts (weighted): proportion (number of participants)

		GUI Cohort 08 (formerly infant cohort)	GUI Cohort 98 (formerly child cohort	MCS	GUS
Gender	Female	48.72% (3989)	48.87% (4404)	48.62% (6823)	48.46% (1528)
	Male	51.28% (4037)	51.13% (4164)	51.38% (7022)	51.54% (1591)
Household composition	Single parent, 1–2 children	10.44% (613)	11.44% (677)	14.63% (1841)	15.04% (377)
	Single parent, 3 + children	4.61% (304)	6.69% (314)	7.96% (1073)	4.67% (98)
	Two parents, 1–2 children	39.7% (3173)	35.06% (3297)	43.35% (6011)	54.97% (1875)
	Two parents, 3 + children	45.25% (3942)	46.81% (4280)	34.07% (4921)	25.32% (769)
Region type	Rural	56.81% (4743)	55.20% (4659)	21.75% (3086)	30.05% (1063)
	Urban	43.19% (3253)	44.80% (3890)	78.25% (10 769)	69.95% (2056)
Household income Quintile	Lowest quintile	20.02% (1154)	19.95% (1055)	20.63% (2852)	28.58% (601)
	Second quintile	20.01% (1355)	20.08% (1375)	20.68% (2860)	19.55% (519)
	Third quintile	19.94% (1433)	20.07% (1583)	20.22% (2796)	18.55% (580)
	Fourth quintile	20.00% (1646)	19.90% (1815)	19.51% (2698)	16.53% (571)
	Highest quintile	20.03% (1704)	20.00% (2114)	18.96% (2621)	16.79% (610)
Special education needs (child)	No	87.71% (7100)	89.42% (7794)	91.15% (12 604)	87.65% (2763)
	Yes	12.29% (926)	10.58% (768)	8.85% (1140)	12.35% (353)

*Note.* Demographics assessed in GUI Cohort ’08 at age 9 (2017), assessed in GUI Cohort ’98 at age 9 (2007), assessed in MCS at age 7 (2008), and GUS at age 8 (2012).

### Statistical analysis

StataMP18 (StataCorp, 2023) and M*plus* (version 8.1; Muthén and Muthén, 2017) were used to calculate descriptive statistics and fit latent growth curve models (LGCM) for child emotional, conduct, and hyperactivity problems in four cohort studies for the full sample and separately by child gender. Participants were included where they had SDQ data for at least one time point. All analyses were conducted using sample-specific weights, stratifications, and clusters (if it is available in the specific cohort) to account for sample attrition and survey design.

LGCMs examined longitudinal changes of mental health difficulties by estimating intercepts, linear slopes and non-linear slopes (quadratic terms) as latent variables. We fitted linear and quadratic curves to the data and compared the fit. Models with better fit were retained. Only linear models could be estimated for GUI cohort ‘08 (formerly infant cohort) and GUI cohort ‘98 (formerly childhood) as each contained only three time points. For each model, the loadings at each time point onto the latent intercept were fixed to a constant (1) and loadings onto the latent growth terms (slope, quadratic terms) were fixed relative to assessment points. All models estimated the association between covariates (household composition; region; income; child SEN), and intercept and growth terms of each youth mental health subscale. Missing data were handled using the full information maximum likelihood (FIML) estimation method (models using complete cases with no missing SDQ data were also fitted as a sensitivity analysis and the patterns revealed no notable differences, please contact the authors for full results). Robust maximum likelihood (MLR) estimation was used to help derive robust standard errors with the presence of nonnormal data. Fit statistics were used to examine model fit using the comparative fit index (CFI), Tucker–Lewis index (TLI), the root mean square error of approximation (RMSEA) and the standardized root mean square residual (SRMR). Good model fit is indicated by a non-significant chi square test, CFI > 0.95, TLI > 0.95, SRMR < 0.08, and RMSEA < 0.06; CFI > 0.90 and TLI > 0.90 indicate acceptable model fit (Hu & Bentler, 1999).

We adhered to the guidelines for strengthening the reporting of observational studies in epidemiology (STROBE) in the reporting of this study (Von Elm et al., 2007; see online Supplementary Table S9 in the supplementary materials). Main codes are available online: https://osf.io/37pa5/.

## Results

### Demographic characteristics

Table 2 outlines core demographic variables within each cohort (further demographics are outlined in online Supplementary Table S1). All cohorts showed similar gender composition and household composition for caregivers. Compared with MCS and GUS, GUI (Cohort ’08 and Cohort ’98) included more participants from rural areas. Children in GUI Cohort ’98 also showed lower rates of special education needs compared with children in GUI Cohort ’08 (see Table 2). All cohorts also showed similar social class and employment status for caregivers. Primary caregivers in GUI Cohort ’98 showed better general health and lower rates of chronic physical or mental illnesses compared with GUI Cohort ’08, MCS, and GUS. Children in GUI Cohort ’98 showed lower rates of chronic illnesses compared with children in GUI Cohort ’08 (see online Supplementary Table S1).

Means and standard deviations of emotional problems, conduct problems, and hyperactivity across cohorts are presented in online Supplementary Table S2 (using sample-specific weights).

### Latent growth curve analyses

LGCMs were estimated for child emotional, conduct, and hyperactivity problems for MCS, GUS, and for GUI Cohort 08 and GUI Cohort 98, conditional upon covariates (see online Supplementary Table S3 and S4). All models showed acceptable or good fit to the data. Removing covariates from the model did not change the pattern of results (see online Supplementary Tables S5–S7).

#### Emotional problems

**Figure 1.**
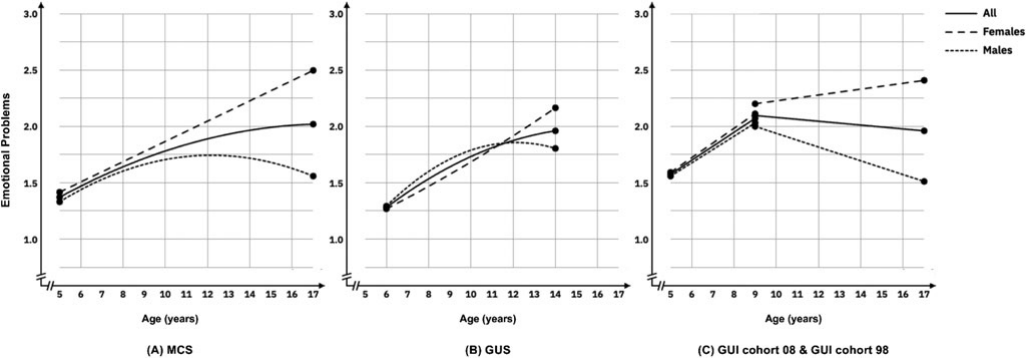
Estimated trajectories of emotional problems from childhood to adolescence in MCS (panel a), GUS (panel b), and GUI Cohorts 08 and 98 (panel c).

The MCS cohort showed a nonlinear trajectory such that emotional problems increased gradually across childhood and adolescence, and then began to decrease in later adolescence. However, when considering trajectories by gender, growth trajectories diverged and gradually widened for boys and girls across development: girls’ emotional problems increased across development, with boys’ emotional problems showing a non-linear trajectory that increased in early childhood and then decreased across later childhood and early adolescence (see Fig. 1 panel a). The GUS cohort also showed a non-linear trajectory with a similar pattern of findings by gender. Growth trajectories diverged for boys and girls from age 12 years. Girls’ emotional problems continued to increase whereas boy's emotional problems began to decrease across later childhood (see Fig. 1 panel b). In GUI cohort 08 (age 5–9 years), boys and girls showed similar intercept and slope, although intercepts were slightly higher at age 5 years in the GUI infancy cohort compared to MCS (age 5 years) and GUS (age 6 years). In the GUI cohort 98 (age 9–17 years) girls’ emotional problems were consistently higher than boys. Boys’ and girls’ trajectories also diverged across adolescence, with girls in GUI (childhood cohort) showing a small but significant increase from age 9 to 17 years whereas boys’ emotional problems declined over this period (see Fig. 1 panel c). The intercept and slope variance of emotional problems were significant in all cohorts, indicating that baseline levels and change rates of youth emotional problems had significant individual differences. Significant negative covariances were observed between the intercept and slope in MCS and GUI cohort 98, indicating that individuals with a higher level of emotional problems at baseline had relatively steeper decreasing or slower increasing trajectories.

Higher income was associated with a lower intercept for boys and girls emotional problems in MCS, GUS and GUI 98 (child cohort) as well as intercept for boys in GUI cohort 08. SEN was associated with a higher intercept of emotional problems for boys and girls across all cohorts. SEN was also associated with steeper emotional problems trajectories for boys and girls in the GUI cohort 08 (age 5–9 years), MCS and GUS (only boys) until mid-adolescence. There were inconsistent associations between region (rural/urban), and family composition and intercept or slope for youth emotional problems across each cohort.

#### Conduct problems

**Figure 2.**
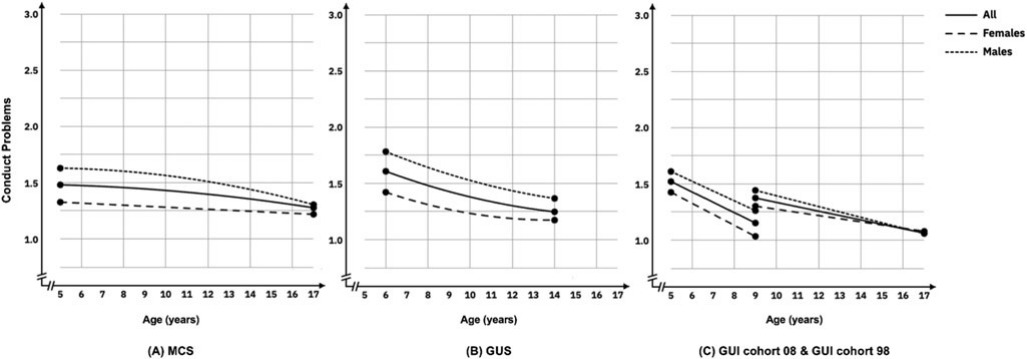
Estimated trajectories of conduct problems from childhood to adolescence in MCS (panel a), GUS (panel b), and GUI Cohorts 08 and 98 (panel c).

Within each cohort, conduct problems were low and decreased across development. Boys’ conduct problems were higher than girls at intercept but gradually converged across development (see Figs 2, panels a–c). With the exception of girls in the GUI cohort 08, the intercept variance and slope variance of conduct problems were significant for all cohorts, indicating that baseline levels and change rate of youth conduct problems had significant individual differences. Significant negative covariances were observed between the intercept and slope in MCS, GUI cohort 98 and boys in GUI cohort 08, indicating that individuals with higher level of conduct problems at baseline have relatively steeper decreasing trajectories.

Higher income was associated with lower conduct problems intercepts for boys and girls across all cohorts with few associations with growth. SEN was associated with higher conduct problems intercept for boys and girls across all cohorts, with some associations with trajectories. There were inconsistent associations between region (rural/urban), and family composition and youth conduct problems intercept or slope across each cohort.

#### Hyperactivity problems

**Figure 3.**
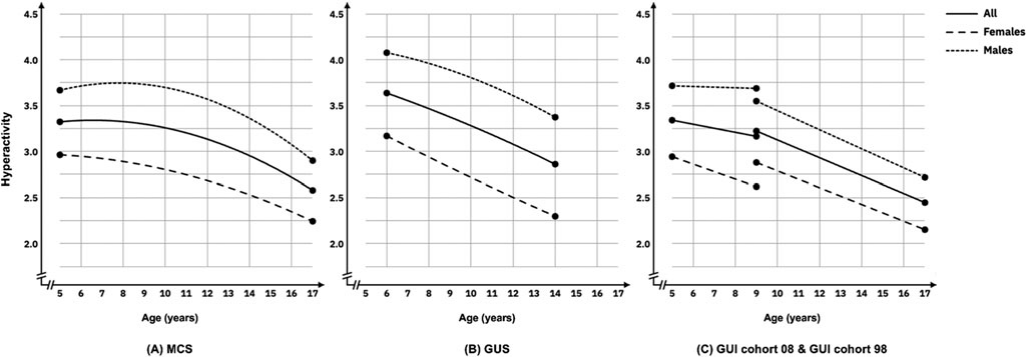
Estimated trajectories of hyperactivity from childhood to adolescence in MCS (panel a), GUS (panel b), and GUI Cohorts 08 and 98 (panel c).

Across all cohorts boys had consistently higher levels of hyperactivity symptoms than girls across development. Hyperactivity symptoms showed non-linear (MCS and GUS cohorts) and linear (GUI infancy and child cohorts) decreases across development, with decreases being similar for boys and girls (see Fig. 3, panels a–c). All cohorts showed significant individual difference in baseline levels and rates of change in hyperactivity symptoms, with the exception of girls in the GUI Cohort 08. Significant negative covariances were observed between the intercept and slope in MCS, GUI cohort 98, and girls in GUS and GUI cohort 08. This indicates that individuals with a higher level of hyperactivity at baseline had steeper decreasing trajectories.

Across all cohorts, higher income was consistently associated with a lower intercept for hyperactivity problems for boys and girls. SEN was associated with higher hyperactivity problems intercept for boys and girls across all cohorts but was inconsistently associated with growth across cohorts. There were also inconsistent associations between region (rural/urban), and family composition and youth conduct problems intercept or slope across each cohort.

## Discussion

This study aimed to examine youth mental health difficulties trajectories (spanning development from ages 5 to 17/18 years) across three representative longitudinal cohorts from the Republic of Ireland, UK, and Scotland. We found that across the three cohorts, there were broadly similar developmental patterns emerging. Conduct problems showed a decreasing trajectory across development and whilst boys’ conduct problems were consistently higher in childhood, boys and girls trajectories converged across development. Hyperactivity problems also showed decreasing trajectories across development, but with boys’ having consistently higher trajectories than girls. Emotional problems were similar for boys and girls in childhood but appeared to diverge across development with girls showing increasing trajectories and boys showing an initial increase, followed by a decrease across adolescence. However, our analyses also suggest that there may be different developmental patterns in the Republic of Ireland (ROI) compared to UK for emotional problems. Findings from estimated means and from trajectory analyses suggest that emotional problems in Ireland emerge earlier than in the UK.

Two potential issues might explain some of the observed differences between cohorts; (i) ability to engage ‘hardly reached’ groups; and (ii) time and societal changes across the different cohorts. To aid planning for services and service development, it is important that cohorts, to be representative, seek to recruit and retain traditionally ‘hardly reached’ groups (e.g. migrant households). Such groups are rarely recruited into cohort studies. GUI data were weighted to match the distribution of the sample to 2006 Census of Population, with the Census having lower representation of some populations (e.g. those from rural areas, migrants). In addition, those with poorer socio-economic status tend to be less likely to participate, and where they do participate, they are less likely to be retained. They are therefore often under-represented in cohort studies. MCS oversampled children from deprived backgrounds and from areas of relatively high ethnic minority concentration in an effort to address this issue. Epidemiological studies should seek to engage hardly reached groups (e.g. migrants, children in the care system) that are not traditionally included to help plan for necessary services. To support engagement with research, there are a number of strategies that have been proposed including researcher training in cultural competence (Erves et al., 2017). Engaging in research-community partnerships by involving participants in research development and research priorities, asking how to accommodate needs, and working with ‘gatekeepers’ (Bonevski et al., 2014; Erves et al., 2017; Nguyen, Palisano, & Graham, 2019) can be helpful. Improving communication regarding research participation opportunities, and expectations (Erves et al., 2017; Nguyen et al., 2019), and using multiple recruitment strategies such as social media and through personal approaches via community groups can also aid engagement (Bonevski et al., 2014; Nguyen et al., 2019). Regular contact between assessment periods can also reduce attrition (David, Alati, Ware, & Kinner, 2013)

The effect of time and key social changes may be relevant to the difference in mental health profiles across the cohorts. For example, the UK and Ireland entered into a recession in 2008 just one year after recruitment of GUI cohorts starting at 9 years/9months, in 2007. In addition, there have been substantial changes over time (e.g. widening health inequalities; lifestyle changes including social media use; Collishaw and Sellers, 2020) which may impact on differences between cohorts. Given such changes, GUI ’08 and ’98 Cohorts may not represent the same population. Caution should be exercised in treating GUI as one accelerated longitudinal cohort design (from 9 months to 17 years). Such an approach is only valid in the absence of cohort effects, as any cohort differences could suggest cultural changes in the cohort/time period (Galbraith, Bowden, & Mander, 2017; Smith, Dawber, & Van Der Heijden, 2019). Demographics were similar between the two GUI cohorts at a household level, suggesting that any cohort effects are unlikely to be explained by differences in household level demographics between cohorts. Nonetheless, this highlights the need for ongoing and up-to-date epidemiological studies to better understand the current mental health needs to support service planning, particularly considering mental health impacts of the Covid-19 pandemic which may impact on the development of youth mental health (Ford, John, & Gunnell, 2021; Kauhanen et al., 2023). Such contexts are not included in the current cohorts.

With estimated increases in mental health prevalence in recent decades, there are increasing calls for reform of services to facilitate change to address identified deficits in access, quality of service provision and outcomes achieved (Finnerty, 2023). It is necessary that we have robust on-going epidemiological data on child and adolescent mental health difficulties (Lynch et al., 2022) to support potential planning, appropriate modeling, and budget allocation. In the absence of reliable epidemiological data, a predictable pitfall for those charged with service resourcing and design is to take waiting lists and service activity as a proxy for population prevalence of need. It is well understood, that waiting lists are an inaccurate reflection of true need, particularly in the case of services seen by the public as inaccessible and ineffective. Furthermore, as demand further heightens and burnout continues, ineffective practices are more likely to propagate as is the likelihood of services employing artificially high criteria to act as barriers to access. As such, to take waiting list/service activity data as a reliable indicator of need is likely to significantly underestimate required resources.

In addition to the points above, other points of reflection should be noted specific to particular strengths and limitations of the present study. Studies chosen were robust methodologically, each reflecting a large cohort, with a national sample and study weights applied to ensure representativeness to general population. For example, there was a high original response rate in GUI (57% child cohort and 81% infant cohort) and a low attrition rate. All datasets used parent-reported SDQ. Whilst it is a methodological strength to use the same measure and reporter for each cohort, evidence suggests that children can report meaningfully on their own mental health (Goodman, Ford, Simmons, Gatward, & Meltzer, 2000; Mathai, Anderson, & Bourne, 2002). Adolescent self-report may be preferred particularly for understanding internal experiences (e.g. emotional problems). Child self-reported SDQ were not available in GUI or MCS (although other measures such as Kessler-6 were available in MCS), but were available in GUS at age 14 years. Results from GUS showed higher levels of both child-reported emotional and behavioral problems compared to parent-reports (see online Supplementary Table S8 for youth self-reported SDQ mean scores at age 14 in GUS). Although the finding in GUS may not generalize to other cohorts (e.g. GUI, MCS), it suggests that parents may not identify some difficulties, and future cohort studies should consider incorporating child self-reports. While each cohort longitudinal study employed relative weights to ensure representativeness of each respective general population, some questions remain regarding the general representativeness of cohort longitudinal studies, particularly in relation to assessing population mental health prevalence rates where the range of problems assessed may be restricted (Keiding & Louis, 2016); an issue of particular significance to mental health service planning. It should also be noted that the original sampling strategies employed by each of the respective longitudinal cohorts included in our manuscript varied in relation to oversampling specific to socioeconomic sample attributes. Specifically, the MCS oversampled children from economically deprived backgrounds, so that the effects of economic disadvantage on children's outcomes could be adjusted and more representatively addressed. GUS and GUI did not oversample children from economically disadvantaged backgrounds, but adjusted the distribution of the respective samples to known population estimates. We cannot eliminate this difference in the respective cohort study designs but highlight this consideration in relation to the interpretation of our derived findings. Notwithstanding these limitations, we highlight the novel use of multiple cohorts to examine our core study questions and the utility of cross-sample comparisons and replication of findings in generating and commending findings to the area of clinical service provision need and related policy decision making.

## Conclusion

In the context of the severe pressure within CAMHS (in the UK and Ireland) and the evident growing demand for services against a backdrop of inadequate resources and available data, it is essential that efforts are made to explore existing datasets to determine to what extent, if any, the data can be effectively utilized to inform clinically meaningful conclusions on population level multi-tiered service needs. Priority must be given to ensure clinically meaningful data is adequately captured in any further cohort studies.

## Supporting information

McNicholas et al. supplementary material McNicholas et al. supplementary material

## Supplementary material

The supplementary material for this article can be found at https://doi.org/10.1017/S0033291724001624.
