# Supplementary material for: Examining the mental health trajectories of children and adolescents: a cross-cohort analysis
Source: Psychol Med. 2024 Nov 20;54(15):4062–70. doi: 10.1017/S0033291724001624 (PMC11650181; doi:10.1017/S0033291724001624)
Supplement: McNicholas et al. supplementary material [file S0033291724001624sup001.docx]

# **Supplementary Materials**

**Examining the mental health trajectories of children and adolescents: A cross-cohort analysis**

Fiona McNicholas, Blanaid Gavin, Ruth Sellers, Iris Ji, Xiaoning Zhang, Wendy V Browne, Gordon Harold

**Table S1**

*Additional Demographics across cohorts (weighted): proportion (number of participants)*

|  |  | GUI Cohort 08 (infant cohort) | GUI Cohort 98 (child cohort | MCS | GUS |
| --- | --- | --- | --- | --- | --- |
| Relationship Status of Primary Caregiver | Married | 73.59% (6252) | 74.91% (6949) | 62.88% (9066) | 62.67% (2164) |
|  | Cohabitating | 10.19% (765) | 6.00% (520) | 12.26% (1514) | 18.45% (480) |
|  | Single | 10.04% (537) | 9.59% (501) | 12.11% (1501) | 12.99% (260) |
|  | Widowed/Divorced | 2.06% (178) | 3.17% (201) | 8.54% (1081) | 2.50% (78) |
|  | Separated | 4.12% (300) | 6.33% (397) | 4.22% (592) | 3.40% (110) |
| Socioeconomic Class (SEC-5) | Managerial and professional occupations |  |  | 28.05% (4024) | 48.20% (1776) |
|  | Intermediate occupations |  |  | 17.53% (2395) | 13.95% (430) |
|  | Small employers and own account workers |  |  | 6.37% (823) | 7.98% (236) |
|  | Lower supervisory and technical occupations |  |  | 4.64% (622) | 7.50% (202) |
|  | Semi-routine and routine occupations |  |  | 34.39% (4643) | 20.92% (455) |
|  | Never worked |  |  | 9.02% (1280) | 1.45% (20) |
| Social Class | Professional workers | 13.40% (1506) | 8.25% (1172) |  |  |
|  | Managerial and technical | 35.58% (3045) | 33.33% (3317) |  |  |
|  | Non-manual | 17.61% (1294) | 18.86% (1686) |  |  |
|  | Skilled manual | 13.35% (899) | 16.60% (1196) |  |  |
|  | Semi-skilled | 9.26% (649) | 9.29% (602) |  |  |
|  | Unskilled | 1.44% (87) | 1.72% (137) |  |  |
|  | All others gainfully occupied and unknown | 1.17% (96) |  |  |  |
|  | Validly no social class | 8.18% (454) | 10.16% (409) |  |  |
|  | Mum & Dad not resident |  | 1.80% (43) |  |  |
| Employment Status (primary caregiver) | In Employment, Education or Training | 64.11% (5541) | 57.82% (5040) | 63.32% (8810) |  |
|  | Unemployed | 2.45% (148) | 2.94% (132) | 1.59% (229) |  |
|  | Looking after family/home | 29.32% (2045) | 39.00% (3329) | 33.34% (4491) |  |
|  | Sick/Disabled |  |  | 1.21% (161) |  |
|  | Other | 4.13% (298) | 0.24% (61) | 0.53% (73) |  |
| Employment Status (primary caregiver) | Full-time (>=35 hrs) |  |  |  | 18.57% (650) |
|  | Part-time (>=16 hrs) |  |  |  | 41.61% (1352) |
|  | Part-time (<16 hrs) |  |  |  | 10.69% (374) |
|  | Not working |  |  |  | 29.12% (739) |
| Employment Status (partner) | In Employment, Education or Training | 92.14% (6574) | 91.09% (6796) | 90.84% (8309) |  |
|  | Unemployed | 5.33% (320) | 4.47% (141) | 2.94% (268) |  |
|  | Looking after family/home | 0.88% (61) | 0.00% (0) | 3.20% (305) |  |
|  | Sick/Disabled |  |  | 2.56% (241) |  |
|  | Other | 1.65% (109) | 4.44% (174) | 0.46% (42) |  |
| Employment Status (partner) | Full-time (>=35 hrs) |  |  |  | 64.23% (2088) |
|  | Part-time (>=16 hrs) |  |  |  | 6.21% (162) |
|  | Part-time (<16 hrs) |  |  |  | 0.54% (18) |
|  | Not working  No partner |  |  |  | 8.08% (171)  20.95% (473) |
| General Health (primary caregiver) | Excellent | 27.77% (2354) | 33.72% (2501) | 22.10% (3130) | 17.40% (591) |
|  | Very good | 39.43% (3264) | 40.40% (2938) | 35.14% (4864) | 36.88% (1210) |
|  | Good | 25.16% (1903) | 20.13% (1368) | 29.63% (4037) | 29.9% (886) |
|  | Fair | 6.54% (437) | 4.90% (265) | 10.24% (1360) | 11.83% (317) |
|  | Poor | 1.20% (71) | 0.85% (44) | 2.89% (374) | 4.00% (97) |
| Chronic illness (primary caregiver) | No | 80.04% (6524) | 90.96% (6523) | 75.01% (10351) | 79.84% (2506) |
|  | Yes | 19.96% (1504) | 9.04% (593) | 24.99% (3408) | 20.16% (596) |
| Chronic Illness or Disability (child) | No | 76.21% (6221) | 88.86% (7698) | 80.86% (11183) | 89.18% (2792) |
|  | Yes | 23.79% (1809) | 11.14% (870) | 19.14% (2580) | 10.82% (326) |
| Ethnicity (primary caregiver) | White | 94.37% (8047) |  | 87.35% (11731) | 96.67% (3035) |
|  | All other ethnic groups | 5.63% (556) |  | 12.65% (2041) | 3.33% (78) |

*Note.* Demographics assessed in GUI Cohort ’08 at age 9 (2017), in GUI Cohort ’98 at age 9 (2007), in MCS at age 7 (2008), and GUS at age 8 (2012). Ethnicity data for GUI Cohort ’08 was extracted from 9-month data (2007).

Additional sociodemographic included here include: relationship status of the primary caregiver (married; cohabiting; single; widowed/divorced; separated), socioeconomic status, employment status of primary and secondary caregivers, general health condition of the primary caregiver, whether the primary caregiver had a long standing illness or disability (yes/no), whether the child had a long standing illness or disability (yes/no), and ethnicity of the child (main caregiver reported ethnicity coded as white; all other ethnic groups) were considered.

Socioeconomic status was assessed in MCS and GUS using the SEC-5 Socioeconomic status. In GUI, seven categories were derived (further information on derivation of SEC categories is available at: <https://www.ons.gov.uk/methodology/classificationsandstandards/otherclassifications/thenationalstatisticssocioeconomicclassificationnssecrebasedonsoc2010> ). Employment status was coded into 5 categories (‘in employment, education or training’; ‘unemployed’’ ‘looking after family/home’; ‘sick/disabled’; ‘other’) in GUI and MCS. In GUS, employment status was derived from questions assessing whether the caregiver was employed, and the number of hours worked.

**Table S2**

*Weighted Mean (Standard Deviation) and Sample Size of Emotional Problems, Conduct Problems and Hyperactivity Scores for Each Sweep of MCS, GUS, GUI Cohort 08, and GUI Cohort 98*

| SDQ | Age (years) |  | Emotional problems | | | Conduct problems | | | Hyperactivity | | |
| --- | --- | --- | --- | --- | --- | --- | --- | --- | --- | --- | --- |
|  |  |  | All | Males | Females | All | Males | Females | All | Males | Females |
| MCS | 5 | Mean | 1.374 | 1.330 | 1.421 | 1.506 | 1.646 | 1.361 | 3.303 | 3.641 | 2.953 |
|  |  | S.D. | (1.588) | (1.585) | (1.590) | (1.510) | (1.590) | (1.407) | (2.379) | (2.451) | (2.250) |
|  |  | N | *14,717* | *7,518* | *7,198* | *14,736* | *7,528* | *7,207* | *14,650* | *7,476* | *7,173* |
|  | 7 | Mean | 1.562 | 1.532 | 1.595 | 1.448 | 1.609 | 1.277 | 3.441 | 3.884 | 2.972 |
|  |  | S.D. | (1.788) | (1.817) | (1.755) | (1.580) | (1.676) | (1.448) | (2.543) | (2.603) | (2.386) |
|  |  | N | *13,433* | *6,826* | *6,605* | *13,462* | *6,840* | *6,620* | *13,412* | *6,819* | *6,591* |
|  | 11 | Mean | 1.907 | 1.844 | 1.973 | 1.456 | 1.617 | 1.287 | 3.236 | 3.704 | 2.742 |
|  |  | S.D. | (2.029) | (2.003) | (2.054) | (1.633) | (1.750) | (1.475) | (2.523) | (2.608) | (2.324) |
|  |  | N | *12,488* | *6,282* | *6,206* | *12,491* | *6,286* | *6,205* | *12,404* | *6,249* | *6,155* |
|  | 14 | Mean | 2.140 | 1.869 | 2.438 | 1.600 | 1.698 | 1.492 | 3.233 | 3.681 | 2.740 |
|  |  | S.D. | (2.202) | (2.064) | (2.314) | (1.777) | (1.857) | (1.665) | (2.514) | (2.577) | (2.330) |
|  |  | N | *11,329* | *5,697* | *5,632* | *11,331* | *5,701* | *5,630* | *11,324* | *5,696* | *5,628* |
|  | 17 | Mean | 2.134 | 1.712 | 2.579 | 1.187 | 1.286 | 1.083 | 2.652 | 3.053 | 2.231 |
|  |  | S.D. | (2.344) | (2.132) | (2.480) | (1.532) | (1.634) | (1.401) | (2.352) | (2.462) | (2.142) |
|  |  | N | *9,271* | *4,646* | *4,623* | *9,275* | *4,648* | *4,625* | *9,268* | *4,644* | *4,622* |
| GUS | 6 | Mean | 1.284 | 1.300 | 1.267 | 1.595 | 1.764 | 1.415 | 3.611 | 4.036 | 3.158 |
|  |  | S.D. | (1.590) | (1.642) | (1.532) | (1.454) | (1.521) | (1.355) | (2.413) | (2.484) | (2.247) |
|  |  | N | *3,357* | *1,719* | *1,638* | *3,360* | *1,721* | *1,639* | *3,356* | *1,720* | *1,636* |
|  | 8 | Mean | 1.550 | 1.628 | 1.466 | 1.548 | 1.735 | 1.348 | 3.557 | 4.098 | 2.979 |
|  |  | S.D. | (1.840) | (1.915) | (1.749) | (1.544) | (1.631) | (1.414) | (2.536) | (2.566) | (2.369) |
|  |  | N | *3,098* | *1,582* | *1,516* | *3,102* | *1,585* | *1,517* | *3,100* | *1,583* | *1,517* |
|  | 10 | Mean | 1.732 | 1.765 | 1.695 | 1.333 | 1.470 | 1.185 | 3.168 | 3.658 | 2.638 |
|  |  | S.D. | (1.985) | (2.035) | (1.925) | (1.490) | (1.571) | (1.379) | (2.485) | (2.555) | (2.287) |
|  |  | N | *3,133* | *1,430* | *1,374* | *3,134* | *1,432* | *1,373* | *3,134* | *1,432* | *1,373* |
|  | 12 | Mean | 2.029 | 2.081 | 1.975 | 1.376 | 1.518 | 1.228 | 3.109 | 3.615 | 2.584 |
|  |  | S.D. | (2.199) | (2.314) | (2.070) | (1.595) | (1.688) | (1.478) | (2.515) | (2.612) | (2.293) |
|  |  | N | *2,492* | *1,260* | *1,232* | *2,497* | *1,264* | *1,233* | *2,494* | *1,262* | *1,232* |
|  | 14 | Mean | 2.023 | 1.839 | 2.212 | 1.267 | 1.333 | 1.185 | 2.868 | 3.352 | 2.373 |
|  |  | S.D. | (2.281) | (2.241) | (2.304) | (1.593) | (1.646) | (1.478) | (2.520) | (2.647) | (2.265) |
|  |  | N | *2,143* | *1,070* | *1,063* | *2,148* | *1,074* | *1,064* | *2,147* | *1,073* | *1,064* |
| GUI  Cohort 08 | 5 | Mean | 1.552 | 1.536 | 1.569 | 1.506 | 1.594 | 1.414 | 3.372 | 3.741 | 2.984 |
|  |  | S.D. | (1.684) | (1.655) | (1.714) | (1.501) | (1.545) | (1.448) | (2.449) | (2.531) | (2.293) |
|  |  | N | *8,998* | *4,565* | *4,433* | *8,999* | *4,566* | *4,433* | *8,996* | *4,565* | *4,431* |
|  | 7/8 | Mean | 1.976 | 1.995 | 1.956 | 1.402 | 1.511 | 1.286 | 3.120 | 3.559 | 2.652 |
|  |  | S.D. | (2.085) | (2.146) | (2.014) | (1.554) | (1.598) | (1.493) | (2.406) | (2.532) | (2.159) |
|  |  | N | *5,281* | *2,668* | *2,613* | *5,282* | *2,669* | *2,613* | *5,278* | *2,666* | *2,612* |
|  | 9 | Mean | 2.097 | 2.075 | 2.121 | 1.178 | 1.293 | 1.057 | 3.251 | 3.757 | 2.719 |
|  |  | S.D. | (2.074) | (2.061) | (2.087) | (1.424) | (1.488) | (1.339) | (2.637) | (2.774) | (2.362) |
|  |  | N | *8,022* | *4,033* | *3,983* | *8.023* | *4,034* | *3,983* | *8,023* | *4,034* | *3,983* |
| GUI  Cohort 98 | 9 | Mean | 2.139 | 2.007 | 2.278 | 1.372 | 1.443 | 1.297 | 3.222 | 3.537 | 2.893 |
|  |  | S.D. | (2.052) | (1.958) | (2.141) | (1.506) | (1.513) | (1.490) | (2.499) | (2.522) | (2.420) |
|  |  | N | *8,560* | *4,158* | *4,402* | *8,551* | *4,156* | *4,395* | *8,549* | *4,156* | *4,393* |
|  | 13 | Mean | 1.904 | 1.716 | 2.098 | 1.228 | 1.245 | 1.210 | 2.815 | 3.160 | 2.457 |
|  |  | S.D. | (2.015) | (1.950) | (2.104) | (1.475) | (1.476) | (1.471) | (2.475) | (2.501) | (2.387) |
|  |  | N | *7,523* | *3,681* | *3,842* | *7,523* | *3,681* | *3,842* | *7,523* | *3,681* | *3,842* |
|  | 17/18 | Mean | 2.017 | 1.507 | 2.547 | 1.041 | 1.041 | 1.041 | 2.430 | 2.691 | 2.160 |
|  |  | S.D. | (2.116) | (1.759) | (2.335) | (1.326) | (1.328) | (1.320) | (2.258) | (2.306) | (2.162) |
|  |  | N | *6,154* | *2,991* | *3,163* | *6,154* | *2,991* | *3,163* | *6,154* | *2,991* | *3,163* |

**Table S3**

*Unstandardized Results and Fit Statistics of Latent Growth Curve Models for Emotional Problems, Conduct Problems and Hyperactivity in MCS, GUS, GUI Cohort 08, and GUI Cohort 98 adjusting for covariates (household income; child SEN; region; family composition)*

|  |  | N | Intercept (I) | | Linear slope (S) | | Quadratic term (Q) | | Correlation (I & S) | Correlation (I & Q) | Correlation (S & Q) | Model fit | | | |
| --- | --- | --- | --- | --- | --- | --- | --- | --- | --- | --- | --- | --- | --- | --- | --- |
|  |  |  | Mean | Variance | Mean | Variance | Mean | Variance |  |  |  | CFI | TLI | RMSEA | SRMR |
| MCS |  |  |  |  |  |  |  |  |  |  |  |  |  |  |  |
| Emotional problems | all | 15148 | 1.371***  (.078) | 1.468***  (.107) | 0.102***  (.004) | .094***  (.009) | -.004***  (.000) | .001***  (.000) | -.065***  (.016) | .001  (.001) | -.006***  (.001) | .998 | .995 | .012 | .006 |
|  | boys | 7743 | 1.330***  (.091) | 1.459***  (.113) | 0.115***  (.006) | .092***  (.013) | -.008***  (.001) | .001***  (.000) | -.052*  (.021) | -.001  (.001) | -.006***  (.001) | .995 | .988 | .018 | .009 |
|  | girls | 7404 | 1.416***  (.066) | 1.466***  (.110) | .090***  (.003) | .093***  (.005) | .000*  (.000) | .001***  (.000) | -.078***  (.014) | .002**  (.001) | -.006***  (.000) | .995 | .989 | .017 | .008 |
| Conduct problems | all | 15150 | 1.480***  (.105) | 1.312***  (.092) | -.005  (.005) | .052***  (.007) | -.001**  (.000) | .000***  (.000) | -.045***  (.008) | -.001  (.001) | -.004***  (.001) | .977 | .948 | .044 | .010 |
|  | boys | 7745 | 1.628***  (.121) | 1.492***  (.091) | -.003  (.006) | .070***  (.007) | -.002***  (.000) | .000***  (.000) | -.058***  (.009) | .000  (.001) | -.005***  (.001) | .991 | .980 | .028 | .008 |
|  | girls | 7404 | 1.326***  (.088) | 1.125***  (.101) | -.009***  (.001) | .008***  (.001) |  |  | -.040***  (.005) |  |  | .953 | .932 | .048 | .022 |
| Hyperactivity | all | 15133 | 3.322***  (.136) | 3.749***  (.105) | .022***  (.005) | .138***  (.010) | -.007***  (.000) | .001***  (.000) | -.144***  (.026) | -.001  (.002) | -.010***  (.001) | .994 | .986 | .026 | .009 |
|  | boys | 7732 | 3.667***  (.143) | 3.954***  (.114) | .056***  (.005) | .150***  (.021) | -.010***  (.001) | .001***  (.000) | -.162***  (.045) | -.002  (.003) | -.010***  (.001) | .989 | .977 | .034 | .010 |
|  | girls | 7400 | 2.962***  (.125) | 3.355***  (.094) | -.012  (.006) | .119***  (.003) | -.004***  (.000) | .001***  (.000) | -.139***  (.015) | -.002  (.001) | -.008***  (.000) | .994 | .987 | .024 | .009 |
| GUS |  |  |  | |  | |  | |  |  |  |  | | | |
|  |  |  |  |  |  |  |  |  |  |  |  |  |  |  |  |
| Emotional problems | all | 3371 | 1.280***  (.033) | 1.554***  (.152) | .141***  (.014) | .198***  (.037) | -.007***  (.002) | .003***  (.001) | -.087  (.060) | .003  (.006) | -.020  (.004) | .999 | .997 | .009 | .007 |
|  | boys | 1730 | 1.292***  (.047) | 1.668***  (.234) | .184***  (.022) | .219***  (.055) | -.015***  (.003) | .003**  (.001) | -.114  (.085) | .005  (.008) | -.023***  (.006) | .996 | .991 | .015 | .011 |
|  | girls | 1641 | 1.268***  (.046) | 1.398***  (.176) | .096***  (.018) | .168***  (.046) | .002  (.002) | .002**  (.001) | -.056  (.073) | .001  (.007) | -.017**  (.005) | 1.000 | 1.004 | .000 | .008 |
| Conduct problems | all | 3371 | 1.607***  (.029) | 1.294***  (.088) | -.069***  (.013) | .107***  (.020) | .003*  (.002) | .002***  (.000) | -.070  (.038) | .001  (.004) | -.011***  (.002) | .992 | .981 | .022 | .011 |
|  | boys | 1730 | 1.782***  (.043) | 1.351***  (.129) | -.076***  (.016) | .113***  (.029) | .003  (.002) | .002***  (.000) | -.054  (.054) | -.001  (.005) | -.012***  (.003) | .992 | .982 | .024 | .012 |
|  | girls | 1641 | 1.421***  (.045) | 1.174***  (.117) | -.063**  (.018) | .098***  (.026) | .004  (.002) | .001***  (.000) | -.087  (.050) | .003  (.005) | -.010***  (.003) | .995 | .989 | .016 | .012 |
| Hyperactivity | all | 3370 | 3.636***  (.039) | 3.946***  (.171) | -.081***  (.018) | .247***  (.031) | -.002  (.002) | .003***  (.000) | -.221***  (.062) | .003  (.006) | -.024***  (.003) | .992 | .983 | .028 | .009 |
|  | boys | 1730 | 4.076***  (.062) | 3.787***  (.288) | -.048  (.025) | .227***  (.048) | -.005  (.003) | .003***  (.001) | -.176  (.100) | .000  (.010) | -.021***  (.005) | .990 | .978 | .033 | .011 |
|  | girls | 1640 | 3.168***  (.069) | 3.795***  (.224) | -.117***  (.023) | .257***  (.046) | .001  (.002) | .003***  (.001) | -.273**  (.087) | .006  (.009) | -.026***  (.005) | .996 | .992 | .018 | .009 |
| GUI cohort 08 | |  |  | |  | |  | |  |  |  |  | | | |
|  |  |  |  |  |  |  |  |  |  |  |  |  |  |  |  |
| Emotional problems | all | 8999 | 1.575***  (.022) | 1.386***  (.111) | .124***  (.007) | .094***  (.014) |  |  | .005  (.029) |  |  | .984 | .951 | .026 | .013 |
|  | boys | 4565 | 1.558***  (.030) | 1.305***  (.158) | .118***  (.010) | .109***  (.020) |  |  | -.012  (.041) |  |  | .982 | .947 | .026 | .016 |
|  | girls | 4434 | 1.592***  (.032) | 1.457***  (.155) | .130***  (.010) | .076***  (.019) |  |  | .021  (.040) |  |  | .990 | .969 | .021 | .011 |
| Conduct problems | all | 9001 | 1.520***  (.019) | 1.094***  (.075) | -.092***  (.005) | .028**  (.008) |  |  | -.038*  (.019) |  |  | .996 | .987 | .014 | .008 |
|  | boys | 4567 | 1.610***  (.028) | 1.288***  (.106) | -.087***  (.007) | .040**  (.012) |  |  | -.057*  (.027) |  |  | .989 | .968 | .024 | .011 |
|  | girls | 4434 | 1.425***  (.026) | .860***  (.105) | -.098***  (.007) | .014  (.011) |  |  | -.014  (.026) |  |  | .995 | .986 | .012 | .011 |
| Hyperactivity | all | 8999 | 3.340***  (.030) | 2.331***  (.151) | -.044***  (.008) | .036  (.019) |  |  | .138**  (.041) |  |  | .997 | .990 | .016 | .009 |
|  | boys | 4566 | 3.715***  (.044) | 2.620***  (.228) | -.007  (.012) | .064*  (.029) |  |  | .114  (.062) |  |  | .997 | .990 | .017 | .011 |
|  | girls | 4433 | 2.942***  (.040) | 1.785***  (.190) | -.081***  (.010) | -.002  (.023) |  |  | .154**  (.049) |  |  | .997 | .990 | .015 | .008 |
| GUI cohort 98 | |  |  | |  | |  | |  |  |  |  | | | |
|  |  |  |  |  |  |  |  |  |  |  |  |  |  |  |  |
| Emotional problems | all | 8566 | 2.096***  (.029) | 2.185***  (.143) | -.017***  (.004) | .023***  (.004) |  |  | -.081***  (.021) |  |  | .978 | .935 | .029 | .012 |
|  | boys | 4163 | 1.999***  (.040) | 2.119***  (.197) | -.061***  (.006) | .023***  (.006) |  |  | -.108***  (.029) |  |  | .999 | .997 | .006 | .008 |
|  | girls | 4403 | 2.200***  (.041) | 2.263***  (.205) | .026***  (.007) | .023***  (.006) |  |  | -.074*  (.030) |  |  | .931 | .793 | .052 | .020 |
| Conduct problems | all | 8565 | 1.374***  (.021) | 1.293***  (.086) | -.039***  (.003) | .014***  (.002) |  |  | -.066***  (.012) |  |  | .997 | .991 | .010 | .007 |
|  | boys | 4164 | 1.442***  (.031) | 1.442***  (.122) | -.048***  (.004) | .015***  (.003) |  |  | -.079***  (.017) |  |  | .996 | .987 | .013 | .009 |
|  | girls | 4401 | 1.302***  (.029) | 1.127***  (.119) | -.028***  (.004) | .012***  (.003) |  |  | -.051**  (.015) |  |  | .996 | .988 | .011 | .007 |
| Hyperactivity/  inattention | all | 8564 | 3.222***  (.035) | 4.124***  (.154) | -.097***  (.005) | .050***  (.004) |  |  | -.226***  (.021) |  |  | .998 | .993 | .012 | .007 |
|  | boys | 4162 | 3.548***  (.051) | 4.384***  (.209) | -.104***  (.007) | .056***  (.007) |  |  | -.243***  (.030) |  |  | .993 | .980 | .021 | .009 |
|  | girls | 4402 | 2.879***  (.047) | 3.572***  (.220) | -.091***  (.006) | .039***  (.006) |  |  | -.191***  (.030) |  |  | .997 | .991 | .013 | .008 |

Note: * *p* < .05; ** *p* < .01; *** *p* < .001

**Table S4**

*Unstandardized Parameters of the Covariates in Latent Growth Curve Models for Emotional Problems, Conduct Problems and Hyperactivity in MCS, GUS, GUI Cohort 08, and GUI Cohort 98*

|  |  | Emotional problems | | | | | | Conduct problems | | | | | | Hyperactivity | | | | | |  |
| --- | --- | --- | --- | --- | --- | --- | --- | --- | --- | --- | --- | --- | --- | --- | --- | --- | --- | --- | --- | --- |
|  |  | Males | | | Females | | | Males | | | Females | | | Males | | | Females | | |  |
|  |  | b | SE | *p* | b | SE | *p* | b | SE | *p* | b | SE | *p* | b | SE | *p* | b | SE | *p* |  |
| MCS |  |  |  |  |  |  |  |  |  |  |  |  |  |  |  |  |  |  |  |  |
| Intercept | Household composition (ref: 2 parents, 1-2 children) | | | | | | |  |  |  |  |  |  |  |  |  |  |  |  |  |
|  | 1 parent, 1-2 children | -.064 | .055 | .243 | .002 | .024 | .938 | **.135** | **.027** | **.000** | **.210** | **.065** | **.001** | **-.162** | **.066** | **.014** | -.037 | .032 | .244 |  |
|  | 1 parent, >3 children | .003 | .050 | .955 | **.336** | **.112** | **.003** | **.376** | **.111** | **.001** | **.372** | **.094** | **.000** | -.007 | .049 | .878 | -.087 | .082 | .286 |  |
|  | 2 parents, >3 children | .013 | .013 | .330 | -.018 | .037 | .626 | .013 | .038 | .737 | **.074** | **.016** | **.000** | **-.268** | **.060** | **.000** | **-.244** | **.022** | **.000** |  |
|  | Income | **-.169** | **.017** | **.000** | **-.160** | **.035** | **.000** | **-.205** | **.013** | **.000** | **-.180** | **.016** | **.000** | **-.308** | **.025** | **.000** | **-.318** | **.023** | **.000** |  |
|  | Urban | **.113** | **.035** | **.001** | .090 | .045 | .044 | **.130** | **.035** | **.000** | **.121** | **.029** | **.000** | **.153** | **.033** | **.000** | **.271** | **.039** | **.000** |  |
|  | Special Education needs | **.631** | **.061** | **.000** | **.590** | **.029** | **.000** | **.833** | **.113** | **.000** | **.636** | **.123** | **.000** | **1.871** | **.101** | **.000** | **1.623** | **.077** | **.000** |  |
| Slope | Household composition (ref: 2 parents, 1-2 children) | | | | | | |  |  |  |  |  |  |  |  |  |  |  |  |  |
|  | 1 parent, 1-2 children | .005 | .015 | .733 | .032 | .017 | .054 | **.135** | **.027** | **.000** | .001 | .005 | .775 | **.111** | **.014** | **.000** | **.145** | **.013** | **.000** |  |
|  | 1 parent, >3 children | .015 | .022 | .481 | **-.058** | **.025** | **.022** | **.376** | **.111** | **.001** | .002 | .005 | .682 | -.047 | .024 | .055 | .051 | .034 | .137 |  |
|  | 2 parents, >3 children | -.018 | .011 | .104 | .001 | .008 | .946 | .013 | .038 | .737 | -.001 | .002 | .768 | .005 | .021 | .796 | **.040** | **.009** | **.000** |  |
|  | Income | -.018 | .011 | .090 | -.005 | .009 | .595 | **-.016** | **.005** | **.001** | -.001 | .001 | .567 | -.009 | .008 | .303 | .002 | .012 | .840 |  |
|  | Urban | .011 | .010 | .269 | -.002 | .014 | .892 | -.009 | .012 | .446 | .004 | .004 | .417 | **-.031** | **.006** | **.000** | .011 | .013 | .370 |  |
|  | Special Education needs | **.145** | **.021** | **.000** | **.101** | **.026** | **.000** | .029 | .019 | .130 | **-.022** | **.008** | **.004** | **.074** | **.026** | **.004** | **.120** | **.033** | **.000** |  |
| Quadratic term | Household composition (ref: 2 parents, 1-2 children) | | | | | |  |  |  |  |  |  |  |  |  |  |  |  |  |  |
|  | 1 parent, 1-2 children | .001 | .002 | .524 | .000 | .001 | .842 | -.001 | .002 | .632 |  |  |  | **-.008** | **.001** | **.000** | **-.010** | **.001** | **.000** |  |
|  | 1 parent, >3 children | -.001 | .002 | .613 | .004 | .002 | .138 | -.001 | .002 | .781 |  |  |  | **.005** | **.002** | **.008** | -.003 | .004 | .454 |  |
|  | 2 parents, >3 children | .001 | .001 | .215 | .000 | .001 | .928 | **-.002** | **.001** | **.000** |  |  |  | .001 | .001 | .409 | -.001 | .001 | .066 |  |
|  | Income | .002 | .001 | .079 | .000 | .001 | .412 | **.002** | **.000** | **.000** |  |  |  | .001 | .001 | .016 | .000 | .001 | .851 |  |
|  | Urban | -.001 | .001 | .377 | .000 | .001 | .959 | .001 | .001 | .508 |  |  |  | **.002** | **.000** | **.000** | -.001 | .002 | .418 |  |
|  | Special Education needs | **-.009** | **.002** | **.000** | **-.007** | **.003** | **.008** | **-.005** | **.001** | **.000** |  |  |  | **-.009** | **.001** | **.000** | **-.012** | **.002** | **.000** |  |
| GUS |  |  |  |  |  |  |  |  |  |  |  |  |  |  |  |  |  |  |  |  |
| Intercept | Household composition (ref: 2 parents, 1-2 children) | | | | | |  |  |  |  |  |  |  |  |  |  |  |  |  |  |
|  | 1 parent, 1-2 children | .289 | .176 | .100 | .271 | .150 | .071 | .081 | .120 | .499 | .280 | .146 | .055 | -.040 | .197 | .841 | .269 | .207 | .192 |  |
|  | 1 parent, >3 children | .577 | .330 | .081 | .476 | .340 | .161 | **.724** | **.339** | **.033** | .584 | .319 | .067 | -.311 | .411 | .450 | -.447 | .481 | .353 |  |
|  | 2 parents, >3 children | .020 | .111 | .860 | .072 | .103 | .482 | .025 | .094 | .787 | .128 | .086 | .133 | **-.347** | **.166** | **.036** | **-.366** | **.148** | **.013** |  |
|  | Income | **-.138** | **.040** | **.001** | **-.077** | **.033** | **.018** | **-.162** | **.031** | **.000** | **-.100** | **.031** | **.001** | **-.301** | **.052** | **.000** | **-.201** | **.047** | **.000** |  |
|  | Urban | **.218** | **.094** | **.020** | -.117 | .099 | .237 | **.209** | **.087** | **.016** | -.034 | .079 | .666 | .315 | .151 | .037 | .201 | .113 | .077 |  |
|  | Special Education needs | **.848** | **.156** | **.000** | **.636** | **.161** | **.000** | **.830** | **.120** | **.000** | **.519** | **.143** | **.000** | **2.239** | **.203** | **.000** | **1.621** | **.246** | **.000** |  |
| Slope | Household composition (ref: 2 parents, 1-2 children) | | | | | |  |  |  |  |  |  |  |  |  |  |  |  |  |  |
|  | 1 parent, 1-2 children | .031 | .088 | .723 | .003 | .071 | .963 | -.060 | .067 | .368 | .006 | .060 | .924 | -.046 | .085 | .586 | -.020 | .087 | .817 |  |
|  | 1 parent, >3 children | -.007 | .152 | .963 | -.105 | .119 | .375 | -.022 | .110 | .838 | -.048 | .128 | .705 | **.407** | **.118** | **.001** | .156 | .155 | .313 |  |
|  | 2 parents, >3 children | **-.117** | **.056** | **.038** | -.012 | .057 | .832 | -.034 | .045 | .446 | **-.066** | **.033** | **.045** | -.083 | .062 | .181 | .058 | .057 | .305 |  |
|  | Income | -.021 | .020 | .286 | -.002 | .014 | .867 | -.024 | .014 | .101 | -.007 | .015 | .615 | -.024 | .021 | .258 | .000 | .017 | .977 |  |
|  | Urban | -.037 | .044 | .399 | -.006 | .039 | .880 | -.033 | .031 | .300 | .040 | .032 | .216 | -.039 | .042 | .352 | -.040 | .045 | .367 |  |
|  | Special Education needs | **.216** | **.058** | **.000** | -.005 | .097 | .960 | .096 | .053 | .073 | -.027 | .083 | .748 | .070 | .075 | .349 | .142 | .083 | .086 |  |
| Quadratic term | Household composition (ref: 2 parents, 1-2 children) | | | | | |  |  |  |  |  |  |  |  |  |  |  |  |  |  |
|  | 1 parent, 1-2 children | -.003 | .011 | .788 | .003 | .009 | .700 | .008 | .008 | .326 | -.003 | .007 | .735 | .006 | .010 | .562 | .009 | .011 | .399 |  |
|  | 1 parent, >3 children | .001 | .020 | .970 | .004 | .014 | .803 | .010 | .015 | .509 | .005 | .015 | .755 | **-.042** | **.013** | **.002** | -.005 | .018 | .783 |  |
|  | 2 parents, >3 children | .011 | .006 | .099 | -.001 | .007 | .843 | .005 | .005 | .366 | **.007** | **.003** | **.047** | .012 | .007 | .088 | -.003 | .007 | .690 |  |
|  | Income | .000 | .003 | .931 | -.002 | .002 | .345 | .003 | .002 | .138 | .000 | .002 | .865 | .001 | .002 | .563 | -.001 | .002 | .497 |  |
|  | Urban | .005 | .005 | .361 | .001 | .005 | .764 | .001 | .004 | .820 | -.004 | .004 | .281 | .001 | .005 | .863 | .000 | .005 | .991 |  |
|  | Special Education needs | **-.022** | **.008** | **.003** | .020 | .012 | .083 | -.013 | .007 | .062 | .010 | .010 | .294 | -.013 | .008 | .129 | -.009 | .012 | .453 |  |
| GUI cohort 08 | |  |  |  |  |  |  |  |  |  |  |  |  |  |  |  |  |  |  |  |
| Intercept | Household composition (ref: 2 parents, 1-2 children) | | | | | |  |  |  |  |  |  |  |  |  |  |  |  |  |  |
|  | 1 parent, 1-2 children | .128 | .150 | .392 | .102 | .164 | .532 | .241 | .150 | .108 | .240 | .122 | .049 | .364 | .182 | .046 | .145 | .186 | .438 |  |
|  | 1 parent, >3 children | -.029 | .217 | .895 | .066 | .177 | .708 | .165 | .195 | .398 | .658 | .226 | .004 | -.038 | .299 | .898 | .348 | .263 | .186 |  |
|  | 2 parents, >3 children | -.083 | .065 | .205 | -.071 | .069 | .300 | -.060 | .060 | .316 | .020 | .056 | .716 | **-.419** | **.091** | **.000** | **-.343** | **.083** | **.000** |  |
|  | Income | **-.105** | **.023** | **.000** | -.036 | .024 | .132 | **-.116** | **.021** | **.000** | **-.104** | **.019** | **.000** | **-.161** | **.031** | **.000** | **-.201** | **.029** | **.000** |  |
|  | Urban | **.147** | **.062** | **.017** | .018 | .064 | .772 | .072 | .057 | .202 | .073 | .053 | .170 | **.215** | **.088** | **.014** | **.243** | **.079** | **.001** |  |
|  | Special Education needs | **.642** | **.102** | **.000** | **.474** | **.134** | **.000** | **.675** | **.090** | **.000** | **.614** | **.119** | **.000** | **1.951** | **.138** | **.000** | **1.693** | **.184** | **.000** |  |
| Slope | Household composition (ref: 2 parents, 1-2 children) | | | | | |  |  |  |  |  |  |  |  |  |  |  |  |  |  |
|  | 1 parent, 1-2 children | .071 | .052 | .169 | .002 | .048 | .962 | -.004 | .034 | .904 | -.037 | .029 | .190 | .028 | .056 | .621 | .047 | .048 | .327 |  |
|  | 1 parent, >3 children | -.039 | .061 | .522 | -.004 | .054 | .945 | .001 | .040 | .982 | -.027 | .052 | .601 | -.062 | .063 | .329 | -.010 | .080 | .900 |  |
|  | 2 parents, >3 children | **-.048** | **.019** | **.013** | -.026 | .020 | .186 | .010 | .015 | .505 | .009 | .014 | .533 | .006 | .023 | .781 | -.004 | .021 | .855 |  |
|  | Income | -.008 | .007 | .313 | -.014 | .007 | .057 | .004 | .005 | .389 | .004 | .005 | .425 | .010 | .008 | .237 | -.001 | .007 | .903 |  |
|  | Urban | -.033 | .020 | .101 | .017 | .019 | .382 | .013 | .015 | .371 | .019 | .014 | .168 | **-.048** | **.023** | **.038** | -.024 | .021 | .260 |  |
|  | Special Education needs | **.264** | **.031** | **.000** | **.293** | **.047** | **.000** | **.070** | **.023** | **.002** | .062 | .032 | .053 | **.330** | **.034** | **.000** | **.251** | **.045** | **.000** |  |
| GUI cohort 98 | |  |  |  |  |  |  |  |  |  |  |  |  |  |  |  |  |  |  |  |
| Intercept | Household composition (ref: 2 parents, 1-2 children) | | | | | |  |  |  |  |  |  |  |  |  |  |  |  |  |  |
|  | 1 parent, 1-2 children | **.508** | **.163** | **.002** | .173 | .159 | .276 | **.279** | **.123** | **.024** | .162 | .107 | .132 | **.714** | **.194** | **.000** | **.545** | **.177** | **.002** |  |
|  | 1 parent, >3 children | -.004 | .213 | .986 | -.174 | .212 | .411 | **.386** | **.195** | **.048** | **.333** | **.149** | **.026** | .466 | .268 | .082 | -.153 | .239 | .521 |  |
|  | 2 parents, >3 children | **-.359** | **.081** | **.000** | **-.321** | **.088** | **.000** | .045 | .062 | .466 | .010 | .063 | .871 | -.198 | .102 | .053 | **-.429** | **.093** | **.000** |  |
|  | Income | **-.111** | **.030** | **.000** | **-.150** | **.032** | **.000** | **-.084** | **.025** | **.001** | **-.128** | **.023** | **.000** | **-.092** | **.038** | **.016** | **-.155** | **.034** | **.000** |  |
|  | Urban | .047 | .078 | .550 | -.018 | .079 | .816 | .039 | .062 | .527 | -.019 | .057 | .745 | .073 | .096 | .450 | -.077 | .089 | .392 |  |
|  | Special Education needs | **1.239** | **.144** | **.000** | **1.086** | **.175** | **.000** | **.903** | **.120** | **.000** | **.535** | **.138** | **.000** | **2.551** | **.166** | **.000** | **2.291** | **.216** | **.000** |  |
| Slope | Household composition (ref: 2 parents, 1-2 children) | | | | | |  |  |  |  |  |  |  |  |  |  |  |  |  |  |
|  | 1 parent, 1-2 children | -.038 | .026 | .144 | -.040 | .026 | .128 | .019 | .017 | .261 | -.021 | .016 | .194 | -.002 | .032 | .955 | **-.079** | **.024** | **.001** |  |
|  | 1 parent, >3 children | .058 | .033 | .080 | -.005 | .036 | .896 | .003 | .025 | .896 | -.030 | .023 | .181 | .031 | .044 | .472 | -.028 | .032 | .383 |  |
|  | 2 parents, >3 children | .009 | .012 | .436 | -.024 | .014 | .084 | .000 | .009 | .965 | -.009 | .009 | .319 | .006 | .014 | .656 | .000 | .013 | .999 |  |
|  | Income | .004 | .004 | .393 | .000 | .005 | .943 | .003 | .003 | .354 | .003 | .003 | .305 | .007 | .006 | .266 | .001 | .005 | .835 |  |
|  | Urban | .020 | .012 | .085 | **.042** | **.013** | **.001** | .005 | .009 | .579 | .014 | .008 | .102 | .006 | .015 | .704 | .022 | .012 | .077 |  |
|  | Special Education needs | -.021 | .022 | .327 | -.026 | .028 | .361 | **-.053** | **.015** | **.001** | -.032 | .019 | .084 | **-.095** | **.023** | **.000** | **-.071** | **.030** | **.019** |  |

**Table S5**

*Unstandardized Parameters of the Covariates in Latent Growth Curve Models for Emotional Problems, Conduct Problems and Hyperactivity in MCS (unadjusted models)*

| SDQ |  | N | Intercept (I) | | Linear slope (S) | | Quadratic term (Q) | | Correlation (I & S) | Correlation (I & Q) | Correlation (S & Q) | Model fit | | | |
| --- | --- | --- | --- | --- | --- | --- | --- | --- | --- | --- | --- | --- | --- | --- | --- |
|  |  |  | Mean | Variance | Mean | Variance | Mean | Variance |  |  |  | CFI | TLI | RMSEA | SRMR |
| Emotional problems | all | 15148 | 1.370***  (.077) | 1.576***  (.128) | 0.102***  (.004) | .097***  (.009) | -.004***  (.000) | .001***  (.000) | -.059**  (.018) | .001  (.001) | -.001***  (.001) | .999 | .999 | .012 | .009 |
|  | boys | 7743 | 1.329***  (.090) | 1.581***  (.125) | 0.114***  (.006) | .098***  (.012) | -.008***  (.001) | .001***  (.000) | -.040  (.023) | -.002  (.001) | -.007***  (.001) | .998 | .997 | .017 | .011 |
|  | girls | 7404 | 1.414***  (.065) | 1.565***  (.140) | .090***  (.003) | .095***  (.005) | .000  (.000) | .001***  (.000) | -.077***  (.018) | .002*  (.001) | -.006***  (.000) | .996 | .993 | .026 | .013 |
| Conduct problems | all | 15150 | 1.479***  (.104) | 1.497***  (.121) | -.006  (.005) | .053***  (.006) | -.001**  (.000) | .000***  (.000) | -.040***  (.009) | -.001*  (.001) | -.004***  (.001) | .979 | .964 | .070 | .018 |
|  | boys | 7745 | 1.627***  (.121) | 1.712***  (.132) | -.005  (.006) | .072***  (.007) | -.002***  (.000) | .000***  (.000) | -.049***  (.011) | -.001  (.001) | -.005***  (.000) | .995 | .991 | .036 | .012 |
|  | girls | 7404 | 1.326***  (.088) | 1.258***  (.115) | -.010***  (.001) | .008***  (.001) |  |  | -.040***  (.006) |  |  | .952 | .952 | .078 | .041 |
| Hyperactivity/  inattention | all | 15133 | 3.320***  (.135) | 4.292***  (.151) | .021***  (.005) | .142***  (.009) | -.007***  (.000) | .001***  (.000) | -.126***  (.033) | -.003  (.002) | -.010***  (.000) | .998 | .996 | .027 | .015 |
|  | boys | 7732 | 3.665***  (.142) | 4.576***  (.156) | .055***  (.005) | .154***  (.019) | -.010***  (.001) | .001***  (.000) | -.149**  (.056) | -.001  (.004) | -.011***  (.001) | .997 | .994 | .032 | .015 |
|  | girls | 7400 | 2.960***  (.124) | 3.734***  (.133) | -.012*  (.006) | .123***  (.003) | -.004***  (.000) | .001***  (.000) | -.123***  (.017) | -.004**  (.001) | -.009***  (.000) | .996 | .993 | .034 | .017 |

Note: * *p* < .05; ** *p* < .01; *** *p* < .001

**Table S6**

*Unstandardized Parameters of the Covariates in Latent Growth Curve Models for Emotional Problems, Conduct Problems and Hyperactivity in GUS (unadjusted models)*

| SDQ |  | N | Intercept (I) | | Linear slope (S) | | Quadratic term (Q) | | Correlation (I & S) | Correlation (I & Q) | Correlation (S & Q) | Model fit | | | |
| --- | --- | --- | --- | --- | --- | --- | --- | --- | --- | --- | --- | --- | --- | --- | --- |
|  |  |  | Mean | Variance | Mean | Variance | Mean | Variance |  |  |  | CFI | TLI | RMSEA | SRMR |
| Emotional problems | all | 3371 | 1.280***  (.033) | 1.700***  (.165) | .141***  (.014) | .204***  (.036) | -.007***  (.002) | .003***  (.001) | -.074  (.062) | .003  (.006) | -.021  (,004) | .998 | .996 | .018 | .011 |
|  | boys | 1730 | 1.292***  (.047) | 1.888***  (.265) | .183***  (.022) | .230***  (.056) | -.016***  (.003) | .003***  (.001) | -.078  (.088) | .002  (.008) | -.024***  (.006) | .990 | .984 | .033 | .020 |
|  | girls | 1641 | 1.267***  (.046) | 1.481***  (.175) | .097***  (.018) | .169***  (.045) | .002  (.002) | .002**  (.001) | -.061  (.072) | .003  (.007) | -.017**  (.005) | 1.000 | 1.004 | .000 | .006 |
| Conduct problems | all | 3371 | 1.607***  (.029) | 1.464***  (.087) | -.070***  (.013) | .112***  (.021) | .003*  (.001) | .002***  (.000) | -.070  (.037) | .001  (.004) | -.012***  (.002) | .990 | .984 | .034 | .016 |
|  | boys | 1730 | 1.782***  (.043) | 1.569***  (.136) | -.078***  (.016) | .116***  (.030) | .003  (.002) | .002***  (.000) | -.036  (.055) | -.003  (.005) | -.013***  (.003) | .990 | .983 | .039 | .018 |
|  | girls | 1641 | 1.421***  (.045) | 1.274***  (.112) | -.063***  (.018) | .105***  (.026) | .004  (.002) | .001***  (.000) | -.098*  (.049) | .005  (.005) | -.011***  (.003) | .994 | .989 | .025 | .015 |
| Hyperactivity/  inattention | all | 3370 | 3.635***  (.040) | 4.721***  (.168) | -.081***  (.018) | .260***  (.033) | -.002  (.002) | .003***  (.000) | -.211**  (.068) | .001  (.007) | -.025***  (.003) | .991 | .985 | .047 | .014 |
|  | boys | 1730 | 4.075***  (.062) | 4.832***  (.311) | -.049*  (.025) | .250***  (.050) | -.005  (.003) | .003***  (.001) | -.168  (.107) | -.002  (.011) | -.024***  (.005) | .987 | .979 | .058 | .016 |
|  | girls | 1640 | 3.168***  (.069) | 4.145***  (.251) | -.115***  (.023) | .259***  (.047) | .001  (.002) | .003***  (.001) | -.270**  (.091) | .007  (.009) | -.026***  (.005) | .995 | .991 | .034 | .013 |

Note: * *p* < .05; ** *p* < .01; *** *p* < .001

**Table S7**

*Unstandardized Parameters of the Covariates in Latent Growth Curve Models for Emotional Problems, Conduct Problems and Hyperactivity in GUI Cohort 08, and GUI Cohort 98 (unadjusted models)*

| GUI 08 (infant cohort) | | | | | | | | | | | |
| --- | --- | --- | --- | --- | --- | --- | --- | --- | --- | --- | --- |
| SDQ |  | N | Intercept (I) | | Linear slope (S) | | Correlation (I & S) | Model fit | | | |
|  |  |  | Mean | Variance | Mean | Variance |  | CFI | TLI | RMSEA | SRMR |
| Emotional problems | all | 8999 | 1.573***  (.022) | 1.476***  (.112) | .122***  (.007) | .114***  (.015) | .012  (.029) | .982 | .947 | .060 | .022 |
|  | boys | 4565 | 1.556***  (.030) | 1.463***  (.160) | .116***  (.010) | .138***  (.021) | -.005  (.042) | .989 | .938 | .060 | .024 |
|  | girls | 4434 | 1.591***  (.032) | 1.493***  (.155) | .128***  (.010) | .088***  (.019) | .030  (.040) | .987 | .962 | .057 | .021 |
| Conduct problems | all | 9001 | 1.518***  (.019) | 1.208***  (.079) | -.093***  (.005) | .031***  (.008) | -.040*  (.019) | .996 | .989 | .028 | .011 |
|  | boys | 4567 | 1.608***  (.028) | 1.437***  (.109) | -.088***  (.007) | .048***  (.012) | -.064*  (.028) | .995 | .985 | .038 | .014 |
|  | girls | 4434 | 1.422***  (.026) | .936***  (.114) | -.098***  (.007) | .013  (.012) | -.012  (.027) | 1.000 | .999 | .008 | .007 |
| Hyperactivity/  inattention | all | 8999 | 3.337***  (.030) | 2.987***  (.167) | -.044***  (.008) | .067**  (.021) | .170***  (.042) | .99*6* | .987 | .042 | .013 |
|  | boys | 4566 | 3.714***  (.044) | 3.421***  (.250) | -.008  (.011) | .111***  (.032) | .147*  (.064) | .998 | .992 | .033 | .010 |
|  | girls | 4433 | 2.935***  (.040) | 2.181***  (.209) | -.082***  (.010) | .009  (.024) | .179***  (.051) | .992 | .977 | .054 | .018 |
| GUI 98 cohort (child cohort) | | | | | | | | | | | |
| SDQ |  | N | Intercept (I) | | Linear slope (S) | | Correlation (I & S) | Model fit | | | |
|  |  |  | Mean | Variance | Mean | Variance |  | CFI | TLI | RMSEA | SRMR |
| Emotional problems | all | 8566 | 2.908***  (.029) | 2.463***  (.152) | -.020***  (.004) | .025***  (.004) | -.095***  (.022) | .979 | .936 | .067 | .023 |
|  | boys | 4163 | 1.999***  (.040) | 2.452***  (.210) | -.063***  (.006) | .025***  (.006) | -.124***  (.031) | 1.000 | 1.001 | .000 | .005 |
|  | girls | 4403 | 2.209***  (.041) | 2.640***  (.170) | .024***  (.006) | .033  (.000) | -.115***  (.016) | .946 | .919 | .082 | .039 |
| Conduct problems | all | 8565 | 1.374***  (.021) | 1.419***  (.095) | -.039***  (.003) | .015***  (.002) | -.075***  (.012) | 1.000 | 1.002 | .000 | .002 |
|  | boys | 4164 | 1.442***  (.031) | 1.590***  (.136) | -.049***  (.004) | .016***  (.003) | -.088***  (.018) | 1.000 | 1.004 | .000 | .000 |
|  | girls | 4401 | 1.302***  (.029) | 1.233***  (.130) | -.029***  (.004) | .013***  (.003) | -.060***  (.016) | 1.000 | 1.002 | .000 | .003 |
| Hyperactivity/  inattention | all | 8564 | 3.223***  (.035) | 5.010***  (.179) | -.099***  (.005) | .055***  (.005) | -.276***  (.023) | 1.000 | 1.001 | .000 | .001 |
|  | boys | 4162 | 3.547***  (.051) | 5.299***  (.241) | -.106***  (.007) | .062***  (.007) | -.298***  (.033) | 1.000 | 1.000 | .005 | .004 |
|  | girls | 4402 | 2.882***  (.047) | 4.309***  (.262) | -.092***  (.006) | .045***  (.006) | -.236***  (.032) | .998 | .995 | .022 | .008 |

Note: * *p* < .05; ** *p* < .01; *** *p* < .001

**Table S8**

*Weighted Mean (Standard Deviation) and Sample Size of Youth Self-reported SDQ Scale Scores in GUS*

| SDQ | Gender |  | Emotional | Conduct | Hyperactivity |
| --- | --- | --- | --- | --- | --- |
| GUS  Age 14  Child | Total | Mean | 3.493 | 1.924 | 3.880 |
|  |  | S.D. | (2.606) | (1.708) | (2.433) |
|  |  | N | *2,058* | *2,058* | *2,058* |
|  | Male | Mean | 2.540 | 2.078 | 3.772 |
|  |  | S.D. | (2.202) | (1.770) | (2.353) |
|  |  | N | *2,613* | *2,613* | *2,612* |
|  | Female | Mean | 4.448 | 1.781 | 3.999 |
|  |  | S.D. | (2.627) | (1.632) | (2.506) |
|  |  | N | *3,983* | *3,983* | *3,983* |

*Note.* GUS: Growing Up in Scotland.

**Table S9**

STROBE Statement—checklist of items that should be included in reports of observational studies.

|  | **Items** | **Recommendation** | **Section** |
| --- | --- | --- | --- |
| **Title and abstract** | 1 | 1. Indicate the study’s design with a commonly used term in the title or the abstract 2. Provide in the abstract an informative and balanced summary of what was done and what was found | Title and Abstract indicate cohort design and Abstract names epidemiological studies.  See Abstract. |
| **Introduction** |  |  |  |
| Background/rationale | 2 | Explain the scientific background and rationale for the investigation being reported | Integrated throughout Introduction but see paragraphs 1-3 for examples. |
| Objectives | 3 | State specific objectives, including any prespecified hypotheses | See last paragraph of Introduction. |
| **Methods** |  |  |  |
| Study design | 4 | Present key elements of study design early in the paper | See Method, 1^st^ paragraph but also Abstract. |
| Setting | 5 | Describe the setting, locations, and relevant dates, including periods of recruitment, exposure, follow-up, and data collection | See Participants & Study Design (1st paragraph, GUI; 2nd paragraph MCS; 3rd paragraph, GUS). |
| Participants | 6 | (*a*) Give the eligibility criteria, and the sources and methods of selection of participants | See Participants & Study Design (1st paragraph, GUI; 2^nd^ paragraph MCS; 3^rd^ paragraph, GUS). |
| Variables | 7 | Clearly define all outcomes, exposures, predictors, potential confounders, and effect modifiers. Give diagnostic criteria, if applicable | See Method, Measures section. |
| Data sources/ measurement | 8* | For each variable of interest, give sources of data and details of methods of assessment (measurement). Describe comparability of assessment methods if there is more than one group | See Method, Measures section. See also Strengths and Limitations section in discussion. All cohorts used comparable measure (SDQ). Time points were not identical but spanned similar developmental periods (see Table 2). |
| Bias | 9 | Describe any efforts to address potential sources of bias | See Method, Statistical Analysis, 1^st^ paragraph. |
| Study size | 10 | Explain how the study size was arrived at | See Supplementary Materials, Table S2. See Method, Statistical Analysis, 1^st^ paragraph |
| Quantitative variables | 11 | Explain how quantitative variables were handled in the analyses. If applicable, describe which groupings were chosen and why | See Method, no transformations were performed. See Method, Statistical Analysis, 1^st^ paragraph. |
| Statistical methods | 12 | (*a*) Describe all statistical methods, including those used to control for confounding | See Method, Statistical Analysis, 2^nd^ paragraph. |
|  |  | (*b*) Describe any methods used to examine subgroups and interactions | See Method, Statistical Analysis, 1^st^ paragraph. |
|  |  | (*c*) Explain how missing data were addressed | See Method, Statistical Analysis, 2^nd^ paragraph. |
|  |  | (*d*) If applicable, describe analytical methods taking account of sampling strategy | See Method, Statistical Analysis, 1^st^ paragraph. |
|  |  | (*e*) Describe any sensitivity analyses | Complete-case analyses were conducted as sensitivity analyses, see Method, Statistical Analysis, 2^nd^ paragraph. |
| **Results** |  |  |  |
| Participants | 13* | (a) Report numbers of individuals at each stage of study—e.g., numbers potentially eligible, examined for eligibility, confirmed eligible, included in the study, completing follow-up, and analysed | See Supplementary Materials, Tables S3, S5 and S7. See cohort websites for more information on eligibility (referred to in Participants & Study Design, 1st paragraph, GUI; 2nd paragraph MCS; 3rd paragraph, GUS). |
|  |  | (b) Give reasons for non-participation at each stage | No exclusion criteria were applied for this study. See Participants & Study Design (1st paragraph, GUI; 2nd paragraph MCS; 3rd paragraph, GUS) for cohort website details for more information regarding reasons for non-participation. |
|  |  | (c) Consider use of a flow diagram | N/A. For more information regarding sampling and survey design of the MCS, GUS and GUI, see cohort websites. |
| Descriptive data | 14* | (a) Give characteristics of study participants (e.g., demographic, clinical, social) and information on exposures and potential confounders | See Results, 1st paragraph, Table 1 and Supplementary Table S1. |
|  |  | (b) Indicate number of participants with missing data for each variable of interest | See Statistical Analysis, 2^nd^ paragraph. See also Supplementary Materials, Tables S3, S5 and S7. |
| Outcome data | 15* | Report numbers of outcome events or summary measures | See Results, 2^nd^ paragraph. |
| Main results | 16 | (a) Give unadjusted estimates and, if applicable, confounder-adjusted estimates and their precision (e.g., 95% confidence interval). Make clear which confounders were adjusted for and why they were included | See Results section (Latent Growth Curve models are estimated conditional upon covariates (see Supplementary Materials, Tables S3 and S4). For unadjusted models, see Tables S5 to S7. |
|  |  | (*b*) Report category boundaries when continuous variables were categorized | N/A |
|  |  | (*c*) If relevant, consider translating estimates of relative risk into absolute risk for a  meaningful time period | N/A |
| Other analyses | 17 | Report other analyses done—e.g., analyses of subgroups and interactions, and sensitivity analyses | See Method, Statistical Analysis, 1^st^ paragraph. See Results section (latent growth curve analyses) for subgroup analyses by gender.  Complete-case analyses were conducted as sensitivity analyses, see Method, Statistical Analysis, 2^nd^ paragraph. |
| **Discussion** |  |  |  |
| Key results | 18 | Summarize key results with reference to study objectives | See Discussion, 1st paragraph. |
| Limitations | 19 | Discuss limitations of the study, taking into accounts sources of potential bias or imprecision. Discuss both direction and magnitude of any potential bias. | See Discussion, Strengths and Limitations section. |
| Interpretation | 20 | Give cautious overall interpretation of results considering objectives, limitations, multiplicity of analyses, results from similar studies, and other relevant evidence. | Integrated throughout Discussion but see also Conclusion. |
| Generalizability | 21 | Discuss the generalizability (external validity) of the study results | See Discussion, Strengths and Limitations. |
| **Other information** |  |  |  |
| Funding | 22 | Give the source of funding and the role of the funders for the present study and, if applicable for the original study on which the present article is based | See Acknowledgements section. |
